# Endothelial cell metabolism: parallels and divergences with cancer cell metabolism

**DOI:** 10.1186/2049-3002-2-19

**Published:** 2014-09-15

**Authors:** Dries Verdegem, Stijn Moens, Peter Stapor, Peter Carmeliet

**Affiliations:** 1 Laboratory of Angiogenesis and Neurovascular link, Vesalius Research Center, Department of Oncology, University of Leuven, Leuven 3000, Belgium; 2 Laboratory of Angiogenesis and Neurovascular link, Vesalius Research Center, VIB, K.U.Leuven, Campus Gasthuisberg, Herestraat 49, box 912, Leuven 3000, Belgium

**Keywords:** Endothelial cell metabolism, Cancer cell metabolism, Angiogenesis, Metabolic pathways, Metabolism, Antiangiogenic therapy, Cancer, Glycolysis

## Abstract

The stromal vasculature in tumors is a vital conduit of nutrients and oxygen for cancer cells. To date, the vast majority of studies have focused on unraveling the genetic basis of vessel sprouting (also termed angiogenesis). In contrast to the widely studied changes in cancer cell metabolism, insight in the metabolic regulation of angiogenesis is only just emerging. These studies show that metabolic pathways in endothelial cells (ECs) importantly regulate angiogenesis in conjunction with genetic signals. In this review, we will highlight these emerging insights in EC metabolism and discuss them in perspective of cancer cell metabolism. While it is generally assumed that cancer cells have unique metabolic adaptations, not shared by healthy non-transformed cells, we will discuss parallels and highlight differences between endothelial and cancer cell metabolism and consider possible novel therapeutic opportunities arising from targeting both cancer and endothelial cells.

## Why target endothelial metabolism?

**Figure 1:**
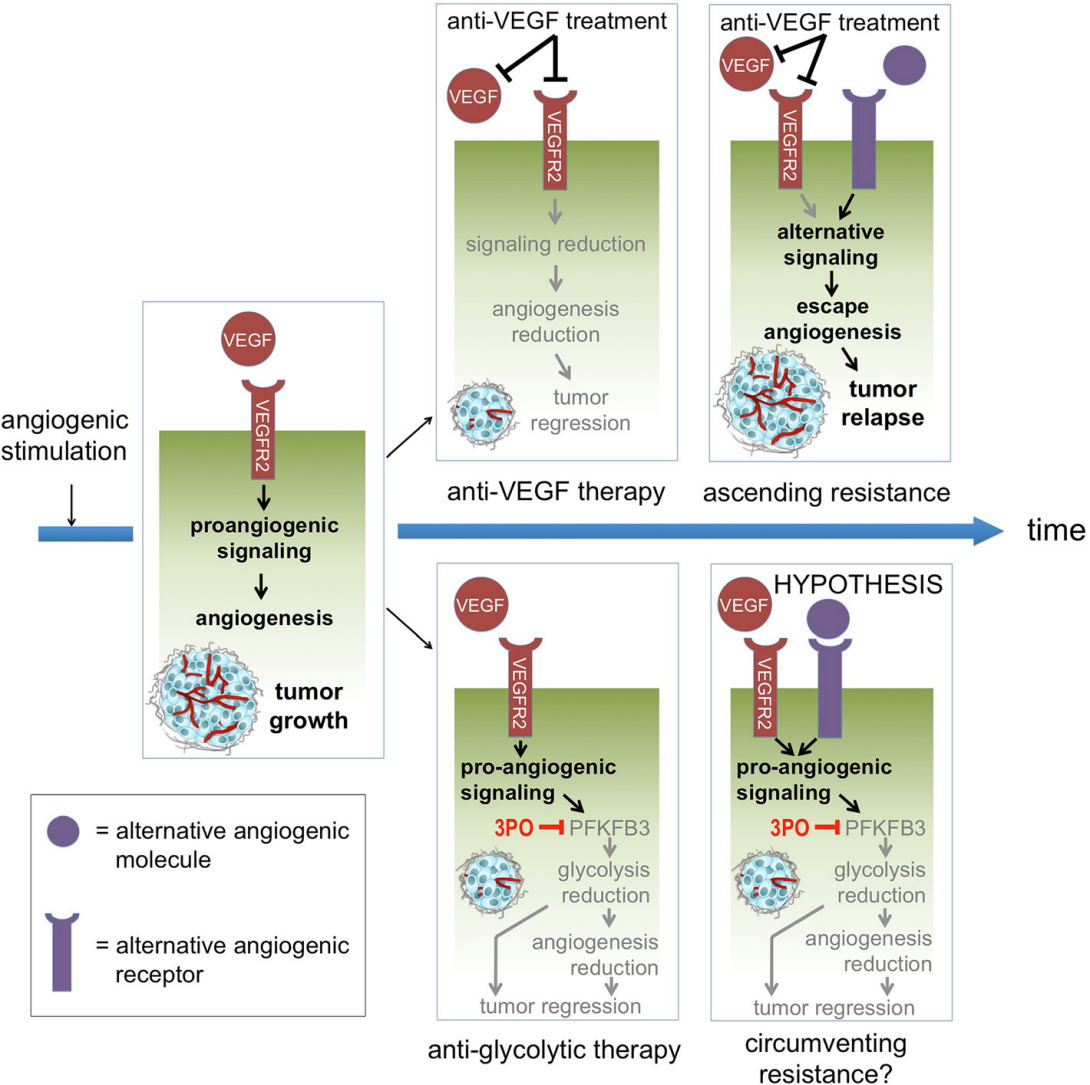
**Rationale to target EC metabolism—a hypothesis.***Figure top*: With time, the conventional anti-VEGF antiangiogenic treatment suffers from increasing resistance due to a shift towards alternative pro-angiogenic molecules of potentially various origins. *Figure bottom*: The antimetabolism treatment bypasses this problem by targeting the PFKFB3 protein downstream of the angiogenic signaling pathways. The blockage of PFKFB3 with 3PO results in a reduction of glycolysis levels and a subsequent halt of the angiogenic process. *3PO* 3-(3-pyridinyl)-1-(4-pyridinyl)-2-propen-1-one, *PFKFB3* 6-phosphofructo-2-kinase/fructose-2,6-bisphosphatase-3, *VEGF* vascular endothelial growth factor, *VEGFR2* vascular endothelial growth factor receptor 2.

Cancer cells are metabolically highly active and require large supplies of nutrients. Blood vessels are vital to their survival, as they not only supply oxygen and nutrients but also remove metabolic waste. Tumors excessively stimulate blood vessel growth to meet their metabolic needs. Thus, a better understanding of how blood vessels nourish tumors can offer novel therapeutic opportunities to prevent or reverse tumor progression. In the past decades, antiangiogenic strategies were primarily based on starving the tumor by destroying the vascular supply through inhibition of key pro-angiogenic molecules. Vascular endothelial growth factor (VEGF) is one of the key regulators of angiogenesis and the prime target of antiangiogenic drug development for the treatment of multiple cancers [1,2]. However, insufficient efficiency and resistance to VEGF-signaling blockade strategies limit their overall success [2-5], necessitating the development of alternative—mechanistically distinct—strategies. We therefore proposed a new paradigm to inhibit tumor growth or other angiogenic pathologies, one that is based on starving the pathological (tumor) vessels themselves from critical metabolic fuel and energy [6,7]. We postulated that when quiescent endothelial cells (ECs) switch to rapid vessel sprouting (the so-called angiogenic switch), they adapt their metabolism to generate additional energy and biomass for growth and division. More simply, the angiogenic switch also requires an ‘angiogenic metabolic switch.’ We further hypothesized that metabolism might be an attractive therapeutic target, since signals from VEGF and other pro-angiogenic factors would centrally converge onto metabolism. In the likely event that cancer cells overcome inhibition of VEGF by upregulating other pro-angiogenic signals to bypass anti-VEGF therapy, metabolism remains the downstream target to increase EC proliferation for angiogenesis (Figure 1). Hence, targeting EC metabolism might offer unprecedented opportunities for the development of alternative antiangiogenic therapies.

In contrast to the vast literature on angiogenesis (nearly 50,000 papers have been published on VEGF alone), only a handful of papers have been published on how ECs adapt their metabolism when shifting from quiescence to rapid growth during vessel sprouting. Most of those papers studied metabolic adaptations of ECs in vitro without any further insight on the importance of such changes for vessel growth in an intact organism or whether they can be targeted for antiangiogenic therapy. Emerging evidence indicates, however, that EC metabolism is an important determinant of EC phenotypes and behavior and a viable target for antiangiogenesis. Before describing our current understanding of EC metabolic adaptations, we will first provide a brief overview on the fundamentals of vessel sprouting.

### Angiogenesis: the basics of vessel sprouting

**Figure 2:**
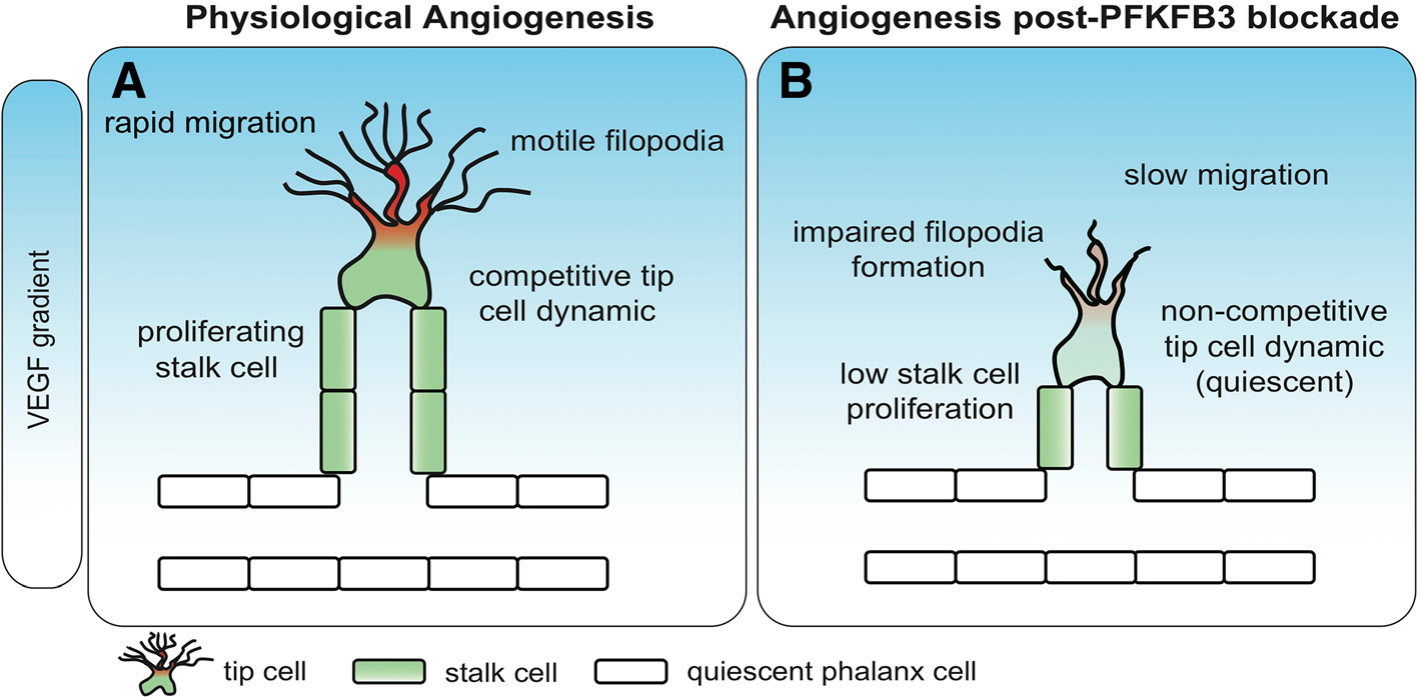
**PFKFB3 blockade reduces angiogenesis. (A)** Current model of vessel sprouting, relying on a migratory tip cell with filopodia that competes for the tip position. Proliferating stalk cells elongate the sprout. Once perfused, ECs turn into quiescent phalanx cells. **(B)** Blocking PFKFB3 results in impaired proliferation and migration of endothelial stalk and tip cells, respectively, and impedes the dynamic overtaking of ECs typically seen during physiological angiogenesis, all resulting in impaired vessel sprouting. Adapted from [157].

**Figure 3:**
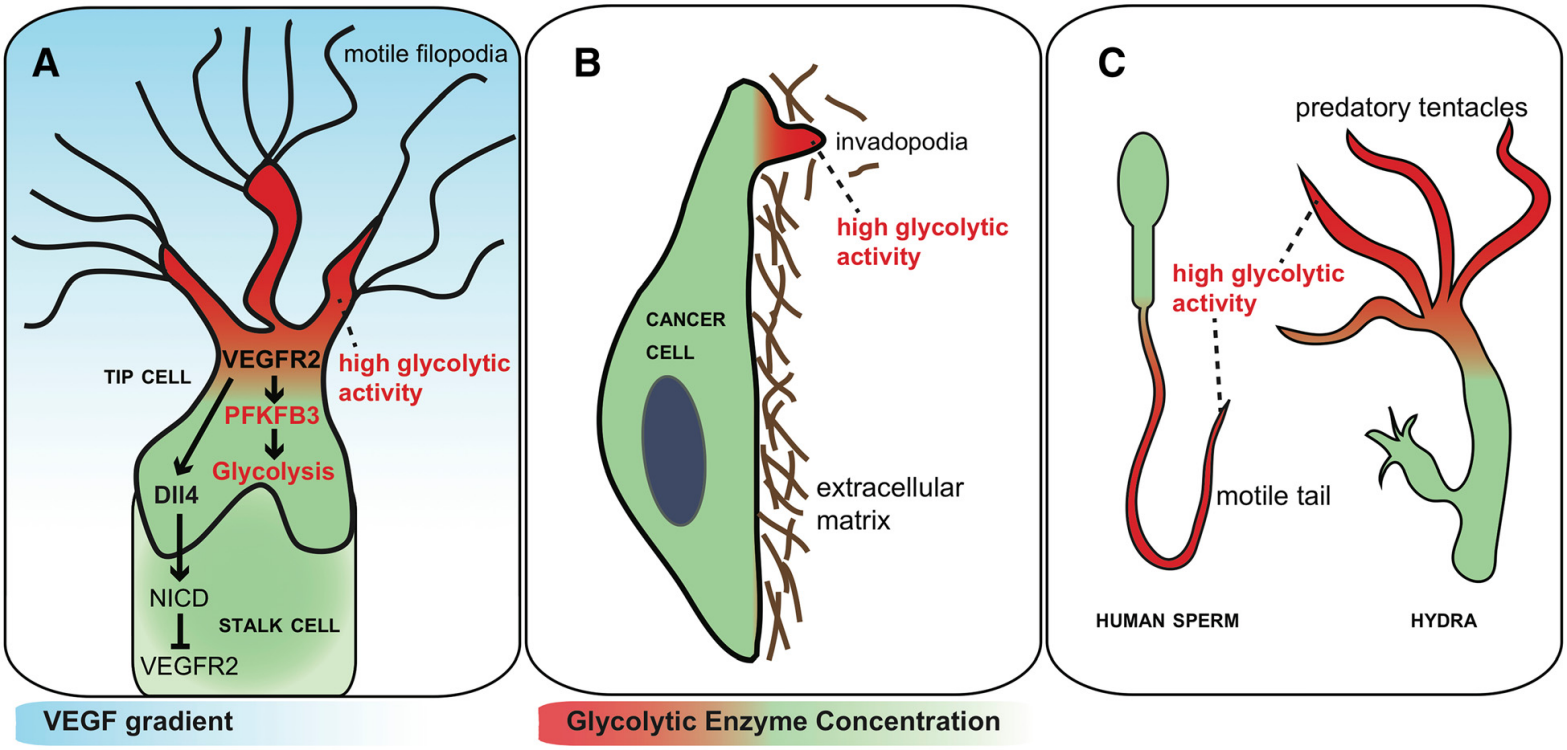
**Glycolysis compartmentalization for cell motility. (A)** Model of lateral inhibition: activation of VEGFR2 in the tip cell by VEGF induces upregulation of Dll4. Activation of Notch on neighboring ECs by Dll4 generates the signaling Notch intracellular domain (NICD), which lowers expression of VEGFR2 and thereby specifies this cell into a stalk cell. Activation of VEGFR upregulates the expression of PFKFB3 and increases glycolysis. Note that glycolytic enzymes and glycolytic ATP production are localized and concentrated in the lamellipodia and filopodia of ECs, where they interact with the actin cytoskeleton. **(B)** In cancer cells, glycolytic enzymes are also concentrated in invasive invadopodia. **(C)** Likewise, glycolytic enzymes are present at higher levels in the motile tail of sperm cells or in motile predatory tentacles of the hydra, suggesting that glycolysis mediates rapid movements of these structures.

In healthy adults, ECs remain quiescent for years. However, these cells can very rapidly start to proliferate and migrate to form new vessels in conditions of injury, inflammation, cancer, or other pathologies. Vessel sprouting in mammals is a highly coordinated process, relying on a migratory (but non- or rarely proliferative) tip cell, and trailing proliferating stalk cells, elongating the sprout shaft (Figure 2A). In the current model (Figure 3A), genetic regulation of angiogenesis involves signaling through VEGFR2, the receptor for the pro-angiogenic factor VEGF [8]. Briefly, VEGFR2 signaling activates the expression of Dll4 on the surface of tip cells. Dll4 is a ligand for the Notch receptor expressed in neighboring stalk cells. Activation of Notch is a potent pro-stalk cell signal in part by downregulation of VEGFR2 and other VEGF coreceptors (like neuropilin-1) and upregulation of VEGF inhibitors (like the VEGF trap VEGFR1) in stalk cells. By activating Notch signaling in neighboring cells, a tip cell thus ensures that it is flanked by stalk cells [9]. In this feedback system, tip cells promote neighboring ECs to assume the stalk cell phenotype in an effect known as lateral inhibition (Figure 3A).

An exciting discovery was that tip and stalk cells are not genetically predetermined, irreversible cell fates, but rather represent plastically changing, reversible EC phenotypes. Tip cells do not remain at the tip for extended periods of time, but continuously shuffle as ECs compete for the position at the tip [10]. Indeed, cells are seen to overtake each other, with cells moving towards or away from the tip [10]. During this movement, cellular VEGFR1 and VEGFR2 levels are continuously re-adjusted to account for the changes in Dll4 expression in neighboring cells. Thus, the tip-stalk cell pattern is a dynamic shuffle among competitive ECs. Until recently, only genetic signals were known to specify the tip versus stalk cell identity, but recent evidence indicates that metabolism is also a key determinant of the EC subtype specification (see below). Whether ECs cannot stay at the tip because they become metabolically exhausted and deprived from nutrients when migrating into an avascular milieu is an intriguing but outstanding question.

During maturation of newly formed vessels, EC secretion of PDGF-B and other signals attracts PDGFR-β expressing pericytes in order to stabilize and functionalize nascent vessels [11]. Mural cell coverage contributes to vessel maturation and induces ECs to once again become quiescent phalanx cells, a process relying on angiopoietin-1/Tie2 signaling, the PHD2 oxygen sensor, junctional molecules (VE-cadherin, claudins, etc.), and other signals [2,12].

The full repertoire of signaling pathways involved in the regulation of angiogenesis is not within the scope of this review and has been reviewed elsewhere [1,8,9,12-14]. Nevertheless, it is worthwhile to emphasize that the complexity of these pathways complicates current attempts to block tumor angiogenesis. For instance, blockage of VEGF evokes escape mechanisms of tumor revascularization by promoting the release of additional pro-angiogenic signals [2-5].

### The tumor microenvironment: a metabolic challenge

The microenvironment in tumors greatly differs from that in healthy tissues. In tumors, vessels are characterized by abnormal structure and function [12,15]. They are dilated, tortuous, and hyperpermeable as caused by irregularities in each of the layers of the vessel wall. ECs are poorly connected and lack a regular pattern. The basement membrane has non-uniform thickness and composition, and mural cells are less abundant and detached. These conditions cause impaired oxygen and nutrient delivery, itself a potent trigger of angiogenesis, thereby leading to a self-reinforcing effect on tumor vessel growth and vascular plexus malformation. For the same reasons, chemotherapy drug delivery is diminished. The defective mural cell coverage also increases the possibility of metastasis [12,15].

Because of its dysfunctional tumor vasculature, cancer cells are deprived of nutrients. Within the tumor interstitium, oxygen is more limiting than glucose. Compared to glucose, oxygen has a higher diffusion coefficient, but lower solubility and therefore also a lower available tissue concentration. The result is a shorter diffusion distance and steeper concentration gradient from the vessel to the tissue [16-18]. As a consequence, cancer and stromal cells alike activate hypoxia signaling through stabilization of HIF-1α, which induces a plethora of adoptive metabolic changes, switching cancer cell metabolism away from oxidative phosphorylation to glycolysis, increasing glycogen synthesis, and shifting from glucose to glutamine as the major substrate for fatty acid synthesis [19,20]. The role of HIFs in adaptive metabolic changes in ECs remains poorly characterized. Nonetheless, which regulatory machinery ECs use to adapt their metabolism remains a relevant question since ECs, unlike many other cell types, must be able to function optimally in oxygen/nutrient-deprived conditions when vascularizing avascular tissues.

A matter of growing importance is to understand how the tumor microenvironment responds to antiangiogenic treatments and the impact this has on the metabolism of cancer and endothelial cells alike. Because current antiangiogenic agents prune tumor vessels, they increase tumor hypoxia and deprive cancer cells from nutrients. As a result, cancer cells turn more to non-oxidative metabolism, which could contribute to the selection of resistant cancer clones [21]. A compelling observation is that antiangiogenic treatment can induce persistent metabolic changes in cancer cells (i.e., suppression of mitochondrial biogenesis and hyperactive glycolysis) [22]. Whether this reflects epigenetic reprogramming of metabolism with long-lasting effects is unknown but certainly deserves investigation. An alternative therapeutic paradigm relies on tumor ‘vessel normalization,’ which restores at least in part the normal structure and function of tumor vessels, thereby improving the supply of oxygen and nutrients [12,15]. Emerging evidence indicates that this lowers glycolytic adaptations of cancer cells [23], but the consequences for EC metabolism still remain unknown.

### Cancer and endothelial metabolism: brothers in arms

Cancer cells and ECs not only reciprocally stimulate each other's growth, but together, they also promote tumor growth. Blood vessels nourish the metabolically demanding cancer cells, while cancer cells enhance metabolism of ECs through releasing pro-angiogenic signals, thereby promoting vessel growth and, in a positive feed-forward loop, also their own growth. This interplay between both cell types provides an attractive target for antitumor strategies. Understanding the metabolic peculiarities of endothelial and cancer cells and the similarities and differences in metabolism between ECs, necessary for tumor growth on one hand, and cancer cells, driving tumor growth on the other hand, may lead to innovative strategies for targeting the factors that fuel aggressive cancer progression. In contrast to the ever-growing knowledge about cancer cell metabolism [24-27], EC metabolism has been studied much less. However, interest in understanding EC metabolism in health and disease is rapidly mounting with innovative technological capabilities and emerging concepts.

**Figure 4:**
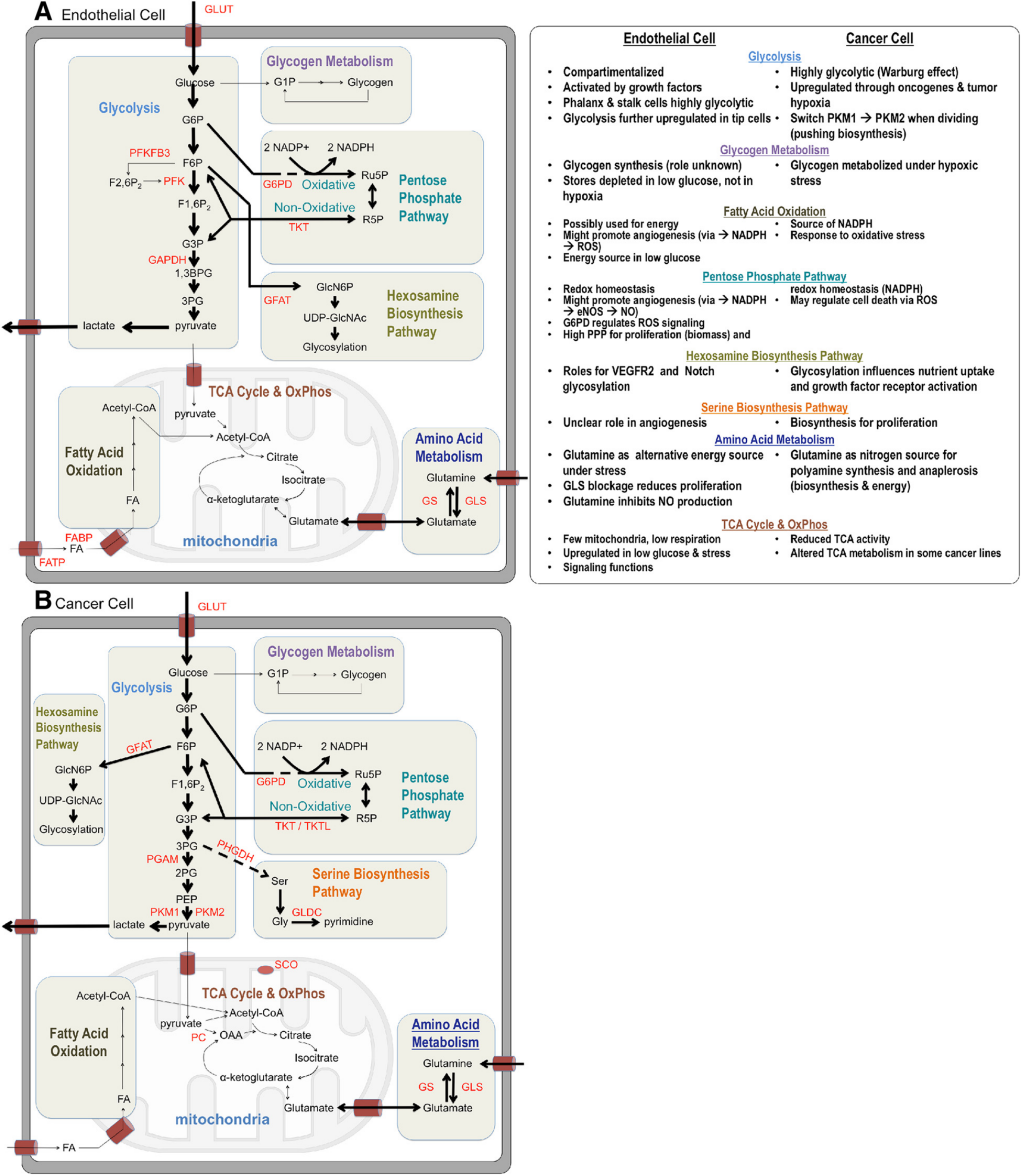
**Endothelial versus cancer metabolism.** Highlights of some common and distinct features of key metabolic pathways displayed by endothelial cells **(A)** and cancer cells **(B)**. *1,3BPG* 1,3-bisphosphoglyceric acid, *2PG* 2-phosphoglycerate, *3PG* 3-phosphoglycerate, *Acetyl-CoA* acetyl-coenzyme A, *eNOS* endothelial nitric oxide synthase, *F1,6P*_*2*_ fructose 1,6 bisphosphate, *F2,6P*_*2*_ fructose 2,6 bisphosphate, *F6P* fructose 6-phosphate, *FA* fatty acid, *FABP* fatty acid binding protein, *FATP* fatty acid transfer protein, *G1P* glucose 1-phosphate, *G3P* glyceraldehyde 3-phosphate, *G6P* glucose 6-phosphate, *G6PD* glucose-6-phosphate dehydrogenase, *GAPDH* glyceraldehyde 3-phosphate dehydrogenase, *GFAT* glutamine-fructose-6-phosphate transaminase, *GlcN6P* glucosamine-6-phosphate, *GLDC* glycine decarboxylase, *GLS* glutaminase, *GLUT* glucose transporter, *Gly* glycine, *GS* glutamine synthetase, *NADP*^*+*^*/NADPH* nicotinamide adenine dinucleotide phosphate, *NO* nitric oxide, *OXPHOS* oxidative phosphorylation, *PC* pyruvate carboxylase, *PEP* phosphoenolpyruvate, *PFK* phosphofructokinase, *PFKFB3* 6-phosphofructo-2-kinase/fructose-2,6-bisphosphatase-3, *PGAM* phosphoglycerate mutase, *PHGDH* phosphoglycerate dehydrogenase, *PK* pyruvate kinase, *PPP* pentose phosphate pathway, *R5P* ribose 5-phosphate, *ROS* reactive oxygen species, *Ru5P* ribulose 5-phosphate, *SCO* cytochrome c oxidase, *Ser* serine, *TCA* tricarboxylic acid, *TKT* transketolase, *TKTL* transketolase-like protein, *UDP-GlcNAc* uridine diphosphate N-acetylglucosamine, *VEGFR2* vascular endothelial growth factor receptor 2.

The recent discovery that vessel sprouting is determined by metabolic regulation (see below) offers novel therapeutic opportunities to synergize with current anticancer therapies or circumvent their shortcomings. Conversely, the recognition that cancer cells adopt their metabolism in response to antiangiogenic treatment and the possibility that cancer cell resistance against antiangiogenic agents may in fact depend on metabolic changes further support the viewpoint that both cell types are closely intertwined and depend on each other [21]. Since cancer cells represent a widely heterogeneous group of cells that rely on multiple types of metabolic adaptations [24-27], even within a single tumor type [28], we will for reasons of brevity not provide an in-depth overview of all possible metabolic alterations in different cancer cell subtypes. Rather, focusing on endothelial metabolism, we will more briefly highlight how metabolism is altered in cancer cells for each of the metabolic pathways discussed in ECs by illustrating key principles and providing selected examples. A schematic of the metabolic pathways discussed and their specifics in endothelial versus cancer cells is illustrated in Figure 4.

### Glycolysis

Most cancer cells are highly glycolytic. Even when sufficient oxygen is available for oxidative glucose metabolism and despite the fact that per mole glucose, much less ATP is generated via glycolysis than glucose oxidation; cancer cells prefer ‘aerobic glycolysis,’ an observation known as the Warburg effect [29,30]. Notably, the tumor suppressor p53, genetically inactivated in many cancers, regulates several metabolic pathways including glycolysis [31]. For instance, p53 suppresses the expression of the glucose transporters GLUT1 and GLUT4 [32] and the glycolytic enzyme phosphoglyceromutase [32,33]. At the same time, p53 not only upregulates TIGAR, an inhibitor of glycolysis that stimulates the oxidative pentose phosphate pathway (oxPPP), but also transactivates cytochrome c oxidase 2 (SCO2), required for the assembly of subunit 2 of the cytochrome c oxidase respiratory complex, essential for mitochondrial respiration [34,35].

In conditions of energy stress, cancer cells use glycolysis for ATP production by relying on pyruvate kinase (PK)-M1, but when they are actively dividing, they switch to PKM2, which diverts glycolytic intermediates into biosynthetic and redox side-pathways (Figure 4B) [36-38]. Indeed, the gene encoding phosphoglycerate dehydrogenase (PHGDH), the rate-limiting enzyme of the serine biosynthesis pathway, is amplified in breast cancers and melanoma [39,40]. Upregulation of PHGDH diverts the glycolytic intermediate 3-phosphoglyceric acid to the serine biosynthesis pathway [40]. Serine can be incorporated into proteins or shuttled into the glycine synthesis pathway and further towards pyrimidine metabolism. Glycine decarboxylase (GLDC), an enzyme upregulated in several cancers, can drive the flux from serine to pyrimidine synthesis [41], but recent evidence indicates that serine, not glycine, supports cancer cell proliferation [42]. Similarly, the PPP and the hexosamine biosynthesis pathway (HBP) use glucose for anabolism and protein glycosylation (see below) [40,43-46].

ECs resemble cancer cells in their preferential use of glycolysis. Endothelial phalanx cells are said to be in a quiescent state because they do not proliferate, and can remain quiescent for years in vivo. From a metabolic point of view, however, this term is misleading as phalanx ECs are anything but metabolically quiescent, and require a substantial baseline glycolysis flux, compared to other quiescent cell types (e.g., quiescent immune cells), in order to function as an endothelium and maintain vascular barrier homeostasis [6]. ECs generate most of their energy through glycolysis [6,47-49] and blocking glycolysis with 2-deoxy-d-glucose (2DG) is toxic for ECs [50,51]. This seems counterintuitive at first, since ECs are in immediate contact with blood, an almost limitless source of oxygen and glucose and thus an ideal environment for oxidative phosphorylation (OXPHOS). ECs are however very plastic cells, and after long-term quiescence, they can start to proliferate rapidly in response to pro-angiogenic stimuli like hypoxia, VEGF, and other signals. We postulated that this angiogenic switch would require a metabolic switch to meet the increased energetic and biosynthetic demands of sprouting ECs to form new blood vessels. Indeed, upon vessel sprouting, ECs nearly double their glycolytic flux [6].

There are several hypothetical reasons why ECs would choose glycolysis over OXPHOS. First, by abstaining from the use of oxygen, ECs preserve the high concentrations of oxygen in the blood for the cells of the tissue they perfuse. Second, oxidative metabolism generates reactive oxygen species (ROS). By using glycolysis, ECs protect themselves and perivascular cells from oxidative stress, in an environment that is already exposed to high levels of oxygen. Third, sprouting ECs migrate through areas of hypoxia. Using an oxygen-independent type of metabolism allows ECs to move from normoxic to hypoxic areas without major adaptations to their metabolism. Indeed, hypoxia is known to induce glycolysis further [52] and upregulate glycolytic enzymes such as glyceraldehyde 3-phosphate dehydrogenase (GAPDH) and others in ECs [53,54]. Fourth, although OXPHOS generates higher amounts of ATP per mole of glucose, glycolysis can actually produce ATP faster, and thus generate comparable amounts of ATP as oxidative metabolism of glucose as long as there is a sufficient supply of glucose [30,55,56]. Thus, a high rate of glycolysis can account for the high metabolic demand necessary for migration and proliferation of ECs during vessel sprouting. A last possible reason why ECs rely on glycolysis could be that glycolysis branches off into macromolecule synthesis pathways. Similar to cancer cells, high glycolysis could allow ECs to maintain these pathways for biomass production and proliferation, necessary for angiogenesis. Pro-angiogenic signaling can indeed regulate the flux through glycolysis, the PPP and glycogen storage in ECs [6,57], but further ^13^C-metabolic isotope labeling studies are required to map the anabolic metabolism of angiogenic ECs.

Phosphofructokinase-2/fructose-2,6-bisphosphatase 3 (PFKFB3) is a stimulator of glycolysis. One of the rate-limiting steps of glycolysis, the conversion of fructose-6-phosphate (F6P) to fructose-1,6-bisphosphate (F1,6P_2_) by 6-phosphofructo-1-kinase (PFK-1) is enhanced by the allosteric coactivator fructose-2,6- bisphosphate (F2,6P_2_), the product of PFKFB3 [6]. PFKFB3 and glycolysis are upregulated by the tip cell signal VEGF and downregulated by the stalk cell signal Notch in ECs [6] (Figure 3A). Hence, glycolysis levels are believed to be higher in tip than stalk cells in the growing vessel sprout, even though glycolysis in stalk cells is also necessary for proliferation of these cells. VEGF also upregulates the expression of facilitated glucose transporter type 1 (GLUT-1), increasing glucose uptake [58]. Thus, it seems that angiogenic signaling factors regulate metabolism, a finding that fits well with the hypothesis that ECs must adapt their metabolism to fuel migration and proliferation during angiogenesis. In an in vitro angiogenesis model, silencing of PFKFB3 reduces glycolysis and vessel sprouting, whereas overexpression of PFKFB3 induces opposite effects [6]. Moreover, EC-specific deficiency of PFKFB3 inhibits vessel growth and causes vascular defects in several in vivo models of angiogenesis. This is due to impaired stalk cell proliferation and tip cell migration (Figure 2B). In fact, PFKFB3 knockdown not only negatively influences tip cell selection, but also abrogates increased tip cell competence upon Notch silencing in vitro [6]. PFKFB3-driven glycolysis can even overrule the potent pro-stalk cell effect of Notch signaling and render stalk cells sufficiently competitive to reach the tip in an in vivo zebrafish model of vessel sprouting. Hence, glycolysis is capable of cospecifying the tip versus stalk cell identity of ECs during sprouting and can overrule genetic angiogenic signals.

Interestingly, when staining for PFKFB3 and other glycolytic enzymes, their presence was localized in the perivascular cytosol in quiescent contact-inhibited ECs, but was also detected at the membrane ruffles in lamellipodia of migrating ECs, i.e., at the very cytoskeletal protrusions that pull ECs forward [6]. PFKFB3 co-stained with F-actin in membrane ruffles of lamellipodia at ‘hotspots’ of ATP generation. These data led to the hypothesis that glycolytic enzymes maintain lamellipodia motor activity necessary for migration by forming ‘glycolytic hubs’, for the local generation of ATP in lamellipodia. Notably, mitochondria are too bulky and therefore excluded from the thin lamellipodia and tiny filopodia. In agreement, inhibition of mitochondrial respiration does not affect vessel sprouting in vitro, suggesting that mitochondrial ATP production is unnecessary for this process [6]. Together, these findings identify glycolysis as an important regulator of angiogenesis, tightly intertwined with genetic angiogenic signals. Like for many cancer cells, the glycolysis addiction of ECs makes this pathway a target for antiangiogenic therapy (see further below) [7,59,60].

Of note, the relationship between glycolysis, cellular cytoskeleton, and motility may be of more general importance than originally expected (Figure 3). Indeed, glycolytic enzymes are also enriched in tumor invadopodia [61-65]. Moreover, glycolytic enzymes have been associated with structures for rapid movement functions such as in hydra tentacles and sperm flagella (Figure 3C) [66,67], and localized glycolytic machinery may supply constant energy, independent of mitochondria, for the persistent movement of vesicles over long distances in axons [68]. In addition, in transgenic fruitflies, failure of glycolytic enzymes to colocalize in actin-rich sarcomeres results in the inability to fly, even though the full complement of active glycolytic enzymes is present in flight muscles [69]. The selective preference of glycolysis and its compartmentalization in structures involved in rapid movements are supported by findings in the hydra that the more slowly advancing movements of its body over the sea floor relies on oxidative metabolism, while the rapid and dynamically changing movements of its tentacles necessary for praying require anaerobic glycolysis.

### Pentose phosphate pathway

Other metabolic pathways are important for cellular processes and may play important roles in regulating angiogenesis, but their role remains understudied. The pentose phosphate pathway (PPP) is a metabolic pathway necessary for redox homeostasis whose starting substrate, glucose-6-phosphate, is a glycolytic intermediate. There are two branches to the PPP. The oxidative PPP (oxPPP) produces NADPH and ribose-5-phosphate (R5P), whereas the non-oxidative PPP (non-oxPPP) only produces R5P in a reversible manner. Glucose-6-phosphate dehydrogenase (G6PD) is a rate-limiting enzyme of the oxPPP, while transketolase (TKT) fulfills this role for the non-oxPPP. Transketolase-like 1 (TKTL1), a poorly studied homologue of TKT, also has transketolase activity [70,71]. NADPH can be used for the conversion of oxidized glutathione (GSSH) to reduced glutathione (GSH) by glutathione reductase. GSH is a key antioxidant in the defense against damaging reactive oxygen species (ROS). NADPH is also required for lipid and nitric oxide (NO) synthesis, whereas R5P is an essential carbon source for ribose production, used for nucleotide synthesis. Thus, the PPP can be used by cells as an anabolic pathway to generate biomass for proliferation or to bolster an antioxidant defense in conditions of oxidative stress [44,46,72,73].

Because the PPP is important for nucleotide synthesis and redox homeostasis, it is typically upregulated in response to oxidative stress or in rapidly proliferating cells, both in healthy tissue and cancer cells. TKTL1 has been suggested as a biomarker for aggressive cancer types and correlates with metastasis [74,75]. It is required for rapid growth and viability of tumor cells [76] and protects against apoptosis induced by growth factor withdrawal, oxidative stress, or cytotoxic therapy, while TKTL1 silencing hinders tumor cell proliferation [76,77]. In vivo, silencing of tumor cell TKTL1 reduces tumor growth, while overexpression of TKTL1 has opposite effects [78,79]. The tumor suppressor p53 binds and inhibits G6PD of the oxPPP; hence, p53 loss in cancer cells would provide them a growth advantage by increased oxPPP-dependent redox buffering or biomass production [80]. It has been suggested that deregulation of the PPP in cancer cells could be causative to tumor progression [81]. Upregulation of key PPP enzymes such as G6PD increases cancer cell proliferation in vitro [82]. Additionally, the PPP may regulate tumor cell death and apoptosis by regulating GSH and ROS levels, and similar associations have been made with increased chemo and radiotherapy resistance [81,83].

For ECs too, there are several indications that the PPP plays a regulatory role in cell behavior and angiogenesis. First, as an important source of NADPH, the oxPPP contributes to synthesis of the pro-angiogenic signal NO by providing NADPH as cofactor for the endothelial NO synthase (eNOS), [84]. In ECs, overexpression of G6PD increases NADPH and NO levels [84]. Therefore, the oxPPP activity may promote angiogenesis through synthesis of NO, which is known to modulate EC function [85,86]. Second, G6PD seems to directly modulate VEGFR2 signaling in a positive manner. Silencing of G6PD decreased VEGF-induced EC proliferation, migration, and tube formation in vitro [87]. In ECs, G6PD association with the membrane is increased upon VEGF stimulation, suggesting a signaling function of G6PD, downstream of VEGF [88]. Moreover, silencing of G6PD reduced the phosphorylation of VEGFR2 and downstream Akt [87]. The translocation of G6PD to the membrane was in part mediated by G6PD phosphorylation by the tyrosine kinase c-src [88]. Upon abolishing G6PD phosphorylation by mutation of the phosphorylation site, VEGF-mediated Akt phosphorylation and EC migration was reduced [88].

The PPP also regulates redox homeostasis in ECs, and thereby can influence cell viability and angiogenesis. First, downregulation of G6PD increases ROS levels in ECs [89], while conversely, overexpression of G6PD in ECs attenuates the ROS response [84], implying that the PPP has a protective function against oxidative stress in ECs, necessary for vessel maintenance and homeostasis. This is especially relevant in diabetes, as one of the effects of high glucose levels on ECs is an increase in oxidative stress. Hyperglycemia-induced ROS in ECs contributes to disorganization of the vasculature and atherosclerosis [90]. Also, hyperglycemia-induced vascular damage is in part mediated by downregulation of G6PD, resulting in a reduction of NADPH levels [91]. A second consequence of the activity of the PPP in redox balance is that it can negatively affect angiogenesis (thus, in contrast to the abovementioned positive effects). Indeed, since ROS can have dual effects on angiogenesis and can mediate pathological angiogenesis [92], it is conceivable that by replenishing GSH, PPP-derived NADPH may negatively influence angiogenesis. This may explain why patients with hereditary G6PD deficiency have increased vascular retinopathy, a complication from diabetes [93]. Overall, most data implicate a pro-angiogenic effect of the PPP, though an angiostatic activity has also been reported, indicating that its effects on angiogenesis can be context dependent.

### Hexosamine biosynthesis pathway

The hexosamine biosynthesis pathway (HBP) produces uridine-diphosphate-*N*-acetylglucosamine (UDP-GlcNAc) used for O- and N-linked glycosylation necessary for normal protein activity [94,95]. Glutamine:fructose-6-phosphate transaminase (GFAT) converts fructose-6-phosphate and glutamine into glucosamine-6-phosphate (GlucN6P) in the HBP rate-limiting reaction. Subsequent reactions require acetyl-CoA and ATP to produce UDP-GlcNAc, which is then used for O- or N-linked glycosylation. Indeed, glycosylation is a common protein modification and has functional implications in nutrient uptake and growth factor receptor activation in cancer cells [96]. Accordingly, the HBP has been suggested as a potential target to modulate cancer cell proliferation [97]. In a pancreatic tumor model, inhibiting the oncogene Kras decreases GFAT expression, HBP flux, and subsequent protein glycosylation [46]. The reduced HBP flux and protein glycosylation, coupled with reduced non-oxPPP flux, lead to decreased tumor volumes, suggesting that heightened HBP flux may be required to maintain cancer cell state [46]. Still, the functional significance of the HBP flux during angiogenesis is not well characterized and the specific endothelial pathways influenced by glycosylation are not well understood.

ECs cultured in high glucosamine concentrations increase protein glycosylation and reduce migration and tube formation [98]. Furthermore, sprouting from cultured aortic rings is impaired under high glucosamine conditions, possibly by Akt glycosylation [98]. Overexpression of O-GlcNAcase, which reduces glycosylation by cleaving the O-GlcNAc modifications [99], increases EC migration and tube formation [98]. Altering N-linked glycosylation has also been suggested to impair EC angiogenic functions in vitro, but continued investigation is necessary for confirmation [50]. By binding the glycosylated regions of VEGFR2, the glycan binding protein galectin-3 increases its angiogenic activity, presumably by promoting VEGFR2 localization to the EC membrane [100]. VEGFR2 glycosylation and binding by galectin-1 is also responsible for continuous VEGFR2-mediated angiogenic activity independent of VEGF after anti-VEGF treatment in refractory tumors. In addition, genetic ablation of glycosyltransferase, an enzyme involved in VEGFR2 glycosylation in ECs, decreases tumor-associated angiogenesis and tumor growth [101]. Another key angiogenic receptor that is glycosylated is Notch, which enhances its angiostatic activity [102]. Current information on the HBP and glycosylation in ECs indicates their importance in regulating both EC activation and inhibition, but the specific pathways and mechanisms involved in angiogenesis are not well characterized.

### Tricarboxylic acid cycle and oxidative phosphorylation

Oxidative phosphorylation (OXPHOS) is an aerobic metabolic process within mitochondria whereby products from the tricarboxylic-acid (TCA) cycle, fatty acid β-oxidation, and amino acid oxidation contribute electrons to the electron transport chain (ETC) to produce ATP from ADP. According to the Warburg hypothesis, cancer cells prefer to generate ATP by metabolizing glucose into lactate rather than breaking down pyruvate through oxidative steps. Generally, the hypoxic tumor milieu stabilizes HIF-1α whose downstream transcriptional targets increase expression of glycolytic enzymes, presumably to shift away from dependence on oxygen consumption [20]. Mutations to mitochondrial DNA in cancer cells may also contribute to the switch towards glycolytic dependence [103]. Nonetheless, some cancers maintain their oxidative capacity, suggesting that cancer cell types exhibit great differences in their TCA and OXPHOS activity in a context-dependent manner [27,104].

In some cases, oncogenic mutations of TCA or ETC genes drive tumorigenesis. Examples include mutations of fumarate hydratase or succinate dehydrogenase, which truncate the TCA cycle, leading to build up of metabolites that increase pro-tumorigenic HIF-1α stabilization through inhibition of the oxygen sensing prolyl hydroxylases [105,106]. Another example is mutant isocitrate dehydrogenase, which generates a so-called oncometabolite called 2-hydroxy-glutarate that may participate in tumor progression [107-109]. However, 2-hydroxy-glutarate and its definitive role in cancer remain incompletely understood. While certain cancer cells have reduced TCA and ETC activity, others show altered TCA activity. In hypoxia, some cancer cell lines exhibit reductive carboxylation of α-ketoglutarate (α-KG) to sustain cell viability [110-113]. Cancer cells also have altered means to replenish the TCA cycle, for instance via pyruvate carboxylase (PC) or enhanced levels of glutaminase (GLS) driven by Myc expression [114,115]. As explained below, stressed cancer cells rely on FAO to produce ATP for survival or NADPH for redox homeostasis to counter high oxidative stress levels in these cells. Mitochondrial dysfunction has also been associated with antiapoptotic cancer cell phenotypes [116]. Support for this association stems from evidence that several mutations in the ETC, which mediates a key apoptotic pathway, contribute to cancer cell survival [117]. Thus, a plethora of alterations in oxidative metabolism contribute to tumorigenesis.

The role of mitochondria in ECs remains open for extensive research. Most ECs (except those of the blood–brain barrier) contain active mitochondria that compose less than 5% of the cell volume, in general far fewer than in other cell types [118-120]. Although glucose oxidation coupled to OXPHOS is at least 20-fold more efficient than glycolysis in producing ATP per mole of glucose and ECs have immediate access to oxygen, less than 1% of pyruvate is oxidized through the TCA cycle by ECs in vitro, while 99% is converted to lactate through glycolysis [6]. This may explain why oxidative pathways contribute only a small amount of ATP for EC function and why blocking the ATP synthase of the ETC by oligomycin did not impair lamellipodia production of ATP and vessel sprouting from EC spheroids [6]. Similar to cancer cells, ECs have a relatively limited amount of oxygen consumption through OXPHOS [6]. In culture, ECs use only about 35% of their total oxidative capacity [121], but can reserve oxidative capacity and increase glucose oxidation for conditions of high metabolic demand or stress, for instance, when glucose is limited [121-123].

Alternatively, aside from their bioenergetic function, EC mitochondria have also been implicated in signaling. Mitochondria-derived ROS, primarily superoxide anion produced through the electron transport chain, and ROS generated by the enzyme NADPH oxidase act as pro-angiogenic signaling molecules at non-toxic concentrations [124,125]. Besides hemodynamic forces and cyclin strain, VEGF increases mitochondria biogenesis and stimulates mitochondrial metabolism and ROS production of ECs in vitro [126-130], suggesting that mitochondrial OXPHOS may play a role in angiogenesis [131]. Indeed, ROS have been shown to mediate VEGFR2 phosphorylation through inactivation of tyrosine phosphatase activity, highlighting the link between redox control and angiogenesis [132-134]. Through inhibition of the PHD oxygen sensors, ROS activate HIFs, which enhance angiogenesis and glycolytic metabolism supporting this process [135]. ROS can also directly activate pro-angiogenic growth factor receptor or other molecules through oxidation of susceptible cysteine residues [134]. Interestingly, ROS-dependent phosphorylation has also been implicated in modulating both in a positive- and negative-manner EC junction stability, thus dependent on the cellular context stimulating angiogenesis or stabilizing vessels [134]. NO, itself a subtype of ROS, also has various pro-angiogenic activities on ECs [85,86,136]. In diabetes, ROS generated by hyperglycemia promotes ligand-independent but Src-dependent phosphorylation of VEGFR2 within the Golgi compartment, leading to muted responses to exogenous VEGF and limiting angiogenic events [137].

Interestingly, glycolysis-derived ATP is in part dedicated to maintaining the EC mitochondrial network [138], presumably because these organelles are essential for EC homeostasis by controlling other processes than metabolism as well. Indeed, mitochondria in ECs mediate Ca^2+^ storage for its use in signaling for a wide range of physiological cell processes [119]. Moreover, mitochondria control an apoptotic pathway in ECs, involved in vessel regression during network remodeling and actively contributing to vascular diseases [139]. In respiring mitochondria, Ca^2+^ flux through the inner mitochondrial membrane (IMM) is mainly driven by the IMM potential (Δ*Ψm*) that establishes a strong electromotive driving force for Ca^2+^ entry. Hence, mitochondrial respiration is vital for Ca^2+^ handling and prevention of apoptosis [119,124].

Still, the role of mitochondria and oxidative metabolism in angiogenesis remains unclear, and the contributions of mitochondrial ROS versus NADPH oxidase-derived ROS is unknown [140]. It also remains unknown how ECs adapt their TCA flow for ATP production, signaling, redox homeostasis, lipid synthesis, or biomass production. Additionally, little is known about mitochondria or their oxidative capacity in tumor-associated ECs. Whether alterations to EC mitochondria contribute to aberrant tumor angiogenesis or a shift in their metabolic status is not known.

### Fatty acid oxidation

Free fatty acids (FFAs) are another energy source that can be solicited by ECs for the generation of ATP [141]. FFAs are released into the blood by lipolysis of triglycerides in the white adipose tissue in times of fasting and can be stored in the form of triglycerides by the same adipocytes of the white adipose tissue at times of high-energy availability [142]. Cancer cells use FFAs as an alternative energy source when glucose is limiting. For instance, during loss of adhesion, glucose-uptake is restricted in cancer cells, and fatty acid β-oxidation (FAO) is activated in order to maintain ATP production [143]. They can also consume fatty acids, derived from circulating lipoprotein particles or supplied by neighboring cancer-associated adipocytes in a cancer-stromal cell metabolic crosstalk [144-146]. In cancer cells, FAO is also an important source of NADPH. FAO produces acetyl CoA, thus fueling the TCA cycle and subsequent citrate production. Citrate can be converted to malate or isocitrate, thus driving malic enzyme or isocitrate dehydrogenase, in both cases generating NADPH [147]. NADPH is important for redox homeostasis and serves as a cofactor in numerous anabolic pathways necessary for biomass production and proliferation.

FFAs can be used as high-energy source by many tissues, especially skeletal and cardiac muscle [148], to which end FFAs are transported across the endothelium. This process is in part regulated by VEGF-B, a VEGF family member whose known role in angiogenesis is limited but not altogether negligent [149]. VEGF-B regulates the expression of fatty acid transport proteins (FATPs) in ECs, and mice lacking VEGF-B showed reduced uptake and accumulation of lipids in perivascular tissue [150]. The presence of lipid droplets has also been reported within ECs [151], and the intracellular fatty acid binding protein 4 (FABP4), important for FFA uptake and trafficking, seems to be regulated by the main pro-angiogenic growth factor VEGF [152]. Both intra and extracellular stores of FFA can be utilized for FAO by ECs [141]. However, reports on the contribution of FAO to energy production in ECs are variable [49,153-157], as this is probably dependent on environmental, genetic, and metabolic cues.

FAO is induced in ECs by activation of the AMPK signaling pathway upon glucose deprivation [141], possibly in an attempt to maintain ATP levels when glycolysis is challenged. Additionally, inhibition of FAO in cancer cells can increase the vulnerability towards oxidative stress [158]. It is however unknown whether ECs use FAO as a source for NADPH via malic enzyme and isocitrate dehydrogenase, as is seen in cancer cells [159]. If so, however, replenishment of the antioxidant GSH by NADPH may affect EC ROS levels, thus influencing angiogenesis.

### Amino acid metabolism

Cancer cells metabolize amino acids for bioenergetic pathways and biosynthesis. Glutamine is a major source of cancer cell nitrogen and anaplerosis to replenish the TCA cycle for biosynthesis [27,160]. Amino acids are also metabolized by ECs, but the functional significance of amino acid metabolism is not understood. Glutamine, also the primary amino acid studied in ECs, can fuel cell proliferation but its contribution to angiogenesis in vivo has not been determined. ECs can not only take up glutamine from the extracellular milieu but can also produce glutamine from glutamate using glutamine synthetase (GS), though the precise reason is unknown [161-163]. In the first step of glutamine metabolism, the conversion of glutamine to glutamate by glutaminase produces α-ketoglutarate for anaplerosis [26]. Although the contribution of ATP derived from glutamine oxidation to EC function is unclear, pharmacological blockade of glutaminase in ECs reduces proliferation and induces senescence [157,164,165]. When glucose metabolism is impaired by oxidative stress, glutamine metabolism contributes more significantly to ATP production [166]. Thus, ECs use glutamine for ATP synthesis depending on the local environmental conditions. Glutamine metabolism in ECs also contributes carbons for synthesis of biomolecules and may be a source of nitrogen for ornithine synthesis, a precursor of mitogenic polyamines [167]. Glutamine also inhibits the production of NO by eNOS [168], and when metabolized to glucosamine, it inhibits oxPPP flux, which reduces availability of the eNOS coenzyme NADPH [165,169]. How glutamine metabolism influences EC sprouting in health or disease has not been described.

### Glycogen metabolism

Cancer cells have been shown to sequester and metabolize glycogen in response to hypoxic stress in order to maintain proliferative capacity [170]. ECs can also accumulate glycogen derived from glucose or push glucose through the glycogen synthesis pathway, but its role in EC function is unknown [57]. Glycogen stores are depleted in ECs under low-glucose conditions, but maintained in hypoxia [57]. Thus, ECs may use glycogen as a backup energy source, perhaps when ECs navigate into glucose-poor tissue regions. Inhibiting glycogen metabolism also reduces EC viability and migration [57]. Still, glycogen metabolism and synthesis in ECs remains under-investigated.

### Endothelial and cancer cell metabolism: different or alike?

The recent surge in data on cancer cell metabolism and the new insights in EC metabolism have brought their general similarities, differences, and unknowns into light (Figure 4).

### Commonalities

Certainly, various cancer phenotypes exhibit a wide range of metabolic characteristics, but the Warburg effect is considered an overarching feature in many cancer cells [25,27,104,160]. Similarly, ECs are highly glycolytic, allowing both cell types to proliferate excessively in the hypoxic tumor milieu. In fact, glycolytic flux is comparable, or even higher in ECs, than in various cancer cell types [6]. Furthermore, both endothelial and cancer cells use glycolytic metabolism for similar cellular mechanisms, i.e., to regulate their invasive and proliferative behaviors [6]. They also seem to compartmentalize glycolysis in protrusive structures mediating actin cytoskeletal rearrangement during cell motility. The reliance of both cell types on glycolysis implies that this pathway is an attractive prospect to target both cell types in the cancer environment (see below).

By contrast, mitochondrial respiration and ATP production seem to be less essential for the proliferation of both glycolytic ECs and most cancer cell types, though for cancer cells, this varies dependent on metabolic features. By mediating redox control, the oxPPP is vital for cellular survival, as is the case for both stressed endothelial and cancer cells [81,84]. The anabolic activity of the PPP for nucleotide synthesis in cancer cells is likely conserved in proliferating ECs, even though this has not been formally demonstrated yet. Lipid synthesis, used for duplication of membranes or production of signaling molecules, is a characteristic of rapidly proliferating endothelial and cancer cells, and inhibiting this process impairs both of their proliferation [171,172]. Unlike cancer cells, which are forced to adapt to the hypoxic tumor milieu that develops around them, ECs are adjusted to functioning in hypoxic environments while vascularizing avascular tissue regions. In anticipation of facing such harsh conditions, ECs store nutrients (glycogen, lipid droplets) intracellularly. Emerging evidence indicates that cancer cells have similar capabilities [144,146].

### Unknowns

For a number of fundamental metabolic pathways, initial evidence indicates that ECs may resemble cancer cells, but available information is insufficient to draw firm conclusions at this stage. For instance, while it is established that glutamine is a major carbon source to replenish the TCA cycle for anabolism in cancer cells [173], only limited evidence exists that ECs may rely on similar mechanisms. Also, the capability of hypoxic cancer cells to utilize their TCA cycle in the reverse direction for reductive carboxylation to synthesize lipids has not been studied in ECs [110,112]. Remarkably, it remains unknown if either cell type uses fatty acid-derived carbons for biosynthesis of macromolecules to sustain cell proliferation.

Other typical cancer cell metabolic adaptations, such as their reliance on serine, glycine, and one-carbon metabolism [42], have only minimally or not at all been examined in ECs. Additionally, the mechanistic details on how glycolytic enzymes (e.g., PKM1 versus PKM2 [174]) regulate the switch between catabolism and anabolism have not been documented in ECs, though the importance of others (e.g., PFKFB3) has been illustrated in both cell types [6,175]. In any case, the differences between these pathways in endothelial and cancer cells remain to be further unraveled before their therapeutic potential becomes apparent.

### Differences

ECs and cancer cells also exhibit differences in their metabolic needs, pathways, and mechanisms. One clear difference is that in many cases, cancer cells reprogram their metabolism hardwire. For instance, due to genetic alterations, cancer cells differ from ECs by switching on certain metabolic pathways (for instance, serine and glycine metabolism) or inactivating others (for instance, mitochondrial TCA cycle genes) [176]. On the contrary, it is unknown if healthy non-transformed ECs with a stable genome activate such pathways during vessel sprouting.

Additionally, stressed cancer cells use FAO for redox protection through production of NADPH and for survival via generation of ATP [147]. In contrast, ECs do not rely on FAO for substantial ATP production [6], and whether they use FAO for redox homeostasis is also unclear. Although both cell types use the PPP, endothelial and cancer cells use it for cell type-specific needs, aside from generating nucleotides, highlighting its versatility. For instance, ECs use the PPP to promote angiogenesis, in part through signaling and NO production, while cancer cells use this pathway to promote sustained proliferation by securing survival through redox fortification. Likewise, the multifaceted effects of the HBP explain why cancer and endothelial cells use this pathway to meet their context-dependent demands, though the active HBP levels indicate the importance of this pathway.

Finally, cancer and endothelial cells differ fundamentally in at least one other important aspect, i.e., their response to nutrient stress or blockade of a critical metabolic pathway. Indeed, nutrient stress that induces quiescence and catabolism in normal cells can be lethal to cancer cells because oncogenic mutations constitutively drive anabolism. Cancer cells are genetically reprogrammed to continuously proliferate and thus constantly require large amounts of nutrients for anabolism, energy production, and redox homeostasis. Hence, if stressed cancer cells cannot mount an adaptive compensatory metabolic shift, their survival becomes limited. This addiction to nutrients renders cancer cells vulnerable to therapeutic interventions. Recent findings indicate, however, that cancer cells may be able to display more flexibility than perhaps anticipated. Indeed, a genetic study showed that cancer cells are capable of switching from anabolic production of biomass for cell division to more catabolic ATP generation for survival in conditions of energy stress [28]. Healthy ECs, by contrast, can plastically switch back and forth between quiescence and proliferation and can resort to quiescence in conditions of nutrient stress or blockade of a critical metabolic pathway. Indeed, reducing glycolysis, which renders rapidly proliferating ECs more quiescent, sufficed to efficiently inhibit pathological angiogenesis without induction of compensatory metabolic adaptations, indicating that induction of quiescence—not necessarily death—of ECs is a viable therapeutic strategy [7,60].

### The link between metabolism and angiogenesis

In light of the metabolic details of ECs provided above and the description of genetic signaling governing angiogenesis, an appealing question that arises is in how far EC metabolism is a determinant of the angiogenic process and the specification of the EC subtype. There are indeed several key observations that hint on an important role for metabolism upstream of the signaling pathways. For instance, upon VEGF activation of ECs, they enhance glycolysis through PFKFB3 upregulation [6]. If this upregulation fails because of PFKFB3 gene knockout or knockdown, there is a strong impetus for ECs to abort the switch towards migrating or proliferating states. This is demonstrated by impaired EC sprouting and filopodia formation in retinal sprouting vessels and reduced lamellipodia formation in cultured ECs [6]. More interesting still, the overexpression of PFKFB3 in mosaic sprouting assays and zebrafish results in the cells adopting the tip cell phenotype, even in the presence of potent pro-stalk Notch signaling (transcriptionally active Notch1 domain NICD overexpression) [6]. This demonstrates that metabolic fitness is an important regulator of endothelial cell specification.

A second example of metabolism implication in EC function is found in the situation of nutrient deprivation, in which case NAD^+^-dependent deacytelase (SIRT1) becomes activated [177,178]. SIRT1 deacetylates NICD causing a reduced NICD stability, making SIRT1 act as a negative regulator of Notch signaling in ECs [179]. As such, SIRT1 was found to be crucial for normal vessel growth, as absence of SIRT1 results in impairment of postnatal neovascularization [180]. Therefore, SIRT1 might act as a promoter of vascularization in nutrient-deprived tissues. Also, SIRT1 is known to deacetylate and inactivate FOXO1, a negative modulator of angiogenesis activated upon nutrient stress [180]. Combined, this suggests that a delicate interplay between SIRT1 and FOXO1 is required to coordinate angiogenesis in situations of EC nutrient deprivation [181].

Furthermore, it has been observed that within a sprouting blood vessel, ECs dynamically rearrange and alternate between leading tip cell and trailing stalk cell positions within the time frame of hours [10,182]. One hypothesis requiring future confirmation is that tip cells, being highly active, deplete their energy stores and need to be replaced by stalk cells with a higher energy profile. Recently, it was shown using computer modeling that tip-stalk shuffling can be explained mechanically by differential cell-cell adhesion and polarized junctional cortex protrusions [183]. The model suggests that both processes are dependent on VEGFR and Notch signaling and parallels the actual in vivo cell adhesion dynamics. However, one can also speculate about underlying or overruling metabolic incentives, since PFKFB3-deficient ECs are found less frequently at the tip position [6].

### Metabolism-based antiangiogenic therapy

Angiogenesis is tightly linked to several pathologies. Best known is the abnormal or excessive angiogenesis contributing to disorders such as inflammation disorders and cancer [8,184]. From these observations, the concepts of pro- and antiangiogenic therapy have grown, in an attempt to restore the occurring angiogenic anomalies. The classical paradigm for antiangiogenic therapies is the blocking of VEGF or its receptors [185]. However, several issues have limited the success of such VEGF (receptor)-inhibitor treatment. Indeed, tumors can rely on other pro-angiogenic factors besides VEGF or on angiogenic molecules produced by associated stromal cells, making the treatment ineffective [2-5]. The sustained hypoxic conditions caused by the treatment can also select for resistant tumor clones. Further mechanisms are switches by the tumors to other forms of vessel growth, besides sprouting angiogenesis, such as the use of existing vessels (cooption), tumor cell differentiation to channels (vascular mimicry), etc. [2-5].

The recent findings on EC metabolism potentially offer novel antiangiogenic therapeutic opportunities. The discovery that genetic silencing of PFKFB3 in vitro and its inactivation in vivo was capable of reducing EC glycolysis and through this decrease vessel sprouting [157], opened up a way towards metabolism-based antiangiogenic therapies. Therefore, the small molecule 3-(3-pyridinyl)-1-(4-pyridinyl)-2-propen-1-one (3PO), a PFKFB3 inhibitor, was tested for its antiangiogenic potential [7]. It was found that 3PO reduces glycolysis in ECs in vitro with 35–40% without making them switch to aerobic respiration since 3PO administration was not accompanied by an increase in oxygen consumption. In vivo glycolysis reduction was also in the order of 35–40% [7]. The effect of 3PO on glycolysis was found to be reversible, as normal sprouting was recovered after removal in vitro or 6 h after administration in vivo. The observed amounts of reduction correspond to the difference in glycolysis activation observed between quiescent and proliferating or migrating ECs [6], and can therefore be thought to significantly impair EC proliferation/migration. In vitro, 3PO indeed considerably increased the fraction of quiescent ECs at the expense of proliferating and migrating ECs. However, unlike more aggressive glycolysis blockers such as 2-deoxy-d-glucose (2DG), which reduces glycolysis to up to 80%, 3PO did not lead to cell death or unhealthy morphology [7]. Notably, the 3PO phenotype was obtained solely because of a metabolic effect as typical tip and stalk cell gene expressions were not altered [7]. In zebrafish embryos, intersomitic vessel sprouting was impaired in a 3PO dose-dependent manner, and after withdrawal of 3PO, the sprouting was reinvigorated. Also, in postnatal mouse retinas of P1-P4 3PO treated pups, EC proliferation was impaired [7].

Moving towards pathological angiogenesis and therapeutic implications, the following observation becomes noteworthy. 3PO administration allowed for preventing to large extent the vascular hyperbranching to occur in VEGFR1 (an inhibitor of vascular branching) knockdown zebrafish embryos. When suboptimal doses of 3PO and suboptimal doses of SU5416, a clinically approved VEGFR tyrosine kinase inhibitor, were administered in combination to zebrafish embryos, the angiogenesis impairment was observed to be larger than those obtained by optimal doses of the inhibitors independently [7]. Finally, 3PO was tested in the context of a few angiogenesis-related pathological models. It was administered to mice in which choroidal neovascularization (CNV), a model for age-related macular degeneration (AMD), was induced by laser burn injury. Daily 3PO provision resulted in a dose-dependent reduction of the CNV lesion volume after 14 days, and its effect was amplified in the case of combined administration with DC101 (an anti-VEGFR2 antibody). Upon hyperoxia-induced capillary depletion in mouse pups (a model for retinopathy of prematurity), 3PO treatment reduced the formation of vascular tufts. Also, in two mouse inflammation models, skin psoriasis, and inflammatory bowel disease, 3PO treatment resulted in a reduced disease severity [7]. Along this line, PFKFB3 targeting was recently extended to tumor implantation models [186]. In PFKFB3 knockout mice, tumor size and weight were significantly decreased as compared to control mice. Importantly, 3PO is also a viable chemotherapeutic agent to block tumor proliferation and represents a two-pronged attack on cancer progression [187] (Figure 3). Together, these findings demonstrate that targeting EC metabolism, and in particular glycolysis and PFKFB3, can be effective as a therapeutic inhibition of angiogenesis.

The main advantage that administration of 3PO provides is its incomplete blocking of glycolysis, since completely blocking glycolysis may have adverse effect [187]. Although 3PO's short half-life and rapid clearance induce only transient effects, it remains a promising candidate for antiangiogenic therapy, alone, or in combination with other anti-VEGF (receptor) inhibitors because the partial and transient reduction of glycolysis suffices to inhibit pathological angiogenesis. This supports a paradigm shift in our traditional thinking that antiglycolytic therapy should block glycolysis completely and permanently. Moreover, the commonalities and differences between endothelial and cancer cell metabolic characteristics represent an opportunity for improving tumor therapeutic strategies.

## Conclusions

The close marriage between cancer and endothelial cells, i.e., cancer cells relying on the vascular supply for metabolic support and growth and ECs relying on cancer cell release of pro-angiogenic signals for metabolism-driven angiogenesis, provides a rationale for targeting both cellular compartments when aiming to block tumor growth. The metabolic similarities characterized so far between both cell types suggest that hitting a common metabolic pathway (like glycolysis) may induce these dual benefits. Furthermore, the differences between endothelial and cancer cell metabolism provide alternative routes to specifically aim at one or the other alone or together. Future success in designing novel antimetabolism therapies requires monitoring isotope labeling through metabolic pathways in an unbiased manner and quantitatively identifying fluxes from the data. In addition, recent reports that metabolism of cancer cells in the culture dish is different, even opposite, to what has been measured in the intact tumor in vivo [59,188], necessitates the challenging characterization of EC metabolism in (tumor) vessels as well. It has been postulated that unique cancer cell-specific metabolic pathways should be preferentially targeted to block cancer growth in order to avoid toxicity on healthy tissues. However, recent findings indicate that pharmacologically blocking a central metabolic pathway like glycolysis can inhibit pathological angiogenesis without inducing systemic effects, as long as glycolysis is only partially and transiently reduced and not completely and permanently blocked [7,60]. These studies illustrate that not only the specific metabolic target is important, but the administration protocols and drug dynamics are critical as well. Another outstanding question is whether targeting EC metabolism should aim to prune tumor vessels with a risk of aggravating tumor hypoxia, increasing invasiveness and metastasis, or rather try to normalize tumor vessels to reduce metastasis and to improve chemotherapy. In either case, concomitantly understanding the dynamics of endothelial and cancer cell metabolism will provide avenues for clinical strategies that more specifically curb excessive cancer growth, while maintaining necessary vascular integrity.

## Competing interests

PC declares being named as inventor on patent applications claiming subject matter related to the results described in this paper. All other authors declare no financial competing interests.

## Authors’ contributions

DV coordinated the literature study and the writing of the manuscript in concert with SM, PS and PC. SM and PS participated in reviewing the literature and drafting the text. DV and PS created the figures, and PS and PC critically reviewed the paper. All authors read and approved the final manuscript.
